# Discovery of a novel AR/HDAC6 dual inhibitor for prostate cancer treatment

**DOI:** 10.18632/aging.202554

**Published:** 2021-02-17

**Authors:** Maojun Zhou, Hao Zheng, Yubin Li, Huichao Huang, Xiaoli Min, Shuyan Dai, Wenqiang Zhou, Zhuchu Chen, Guangyu Xu, Yongheng Chen

**Affiliations:** 1 Department of Oncology, NHC Key Laboratory of Cancer Proteomics, State Local Joint Engineering Laboratory for Anticancer Drugs, National Center for Geriatrics Clinical Research, Xiangya Hospital, Central South University, Changsha 410008, Hunan, China; 2 Key Laboratory of Chemical Biology and Traditional Chinese Medicine, Ministry of Educational of China, Key Laboratory of the Assembly and Application of Organic Functional Molecules of Hunan Province, Hunan Normal University, Changsha 410081, Hunan, China; 3 Zeta Pharma, Changsha 410000, Hunan, China

**Keywords:** androgen receptor, HDAC6, prostate cancer, dual inhibitor, Zeta55

## Abstract

Androgen receptor (AR) and histone deacetylase 6 (HDAC6) are important targets for cancer therapy. Given that both AR antagonists and HDAC6 inhibitors modulate AR signaling, a novel AR/HDAC6 dual inhibitor is investigated for its anticancer effects in castration-resistant prostate cancer (CRPC). Zeta55 inhibits nuclear translocation of AR and suppresses androgen-induced PSA and TMPRSS2 expression. Meanwhile, Zeta55 selectively inhibits HDAC6 activity, leading to AR degradation. Zeta55 reduces the growth of AR-overexpressing VCaP prostate cancer cells both *in vitro* and in a CRPC xenograft model. These results provide preclinical proof of principle for Zeta55 as a promising therapeutic in prostate cancer treatment.

## INTRODUCTION

Castration-resistant prostate cancer (CRPC) is the second leading cause of cancer death among men in the United States (31,620 deaths in 2019) [1]. Androgen receptor (AR), a member of nuclear receptor family, plays vital roles in the development of prostate cancer [2]. Although prostate cancer can be stabilized or regressed by androgen deprivation therapy, reactivation of AR by overexpression, point mutation, alternative splicing and other ligand-independent manners ultimately lead to incurable castration-resistant prostate cancer (CRPC) [3]. The competitive non-steroidal AR antagonist MDV3100 (Enzalutamide), which was approved by FDA, strongly improve metastasis-free survival in non-metastatic CRPC [4–6]. However, acquired resistance to AR antagonist MDV3100 eventually results in lethality to metastatic CRPC patients [3], suggesting that better treatment options are still urgently needed for treating CRPC.

Histone deacetylases (HDACs) are involved in epigenetic regulation of gene expression and protein activity by removing acetyl groups from histone or nonhistone proteins [7]. It has been widely recognized that HDACs are clinically validated cancer targets, and histone deacetylase inhibitors (HDACi) can inhibit cancer cell proliferation through apoptosis, autophagy or necrosis [8]. Four HDACi, Vorinostat (SAHA), Romidepsin, Panobinostat and Belinostat, have been approved by FDA for the treatment of hematological malignancies [9]. However, the efficacy of HDAC inhibitors in solid tumors has not been satisfactory partially due to their side effects and toxicity [10].

HDAC6 is primarily localized in the cytoplasm and deacetylates a variety of cytoplasmic proteins, which are involved in protein degradation, protein trafficking, cell migration, and metastasis [11]. Overexpression of HDAC6 is associated with the development of multiple cancers. In recent years, HDAC6-selective inhibitors have become an emerging class of cancer therapeutics, and several HDAC6-selective inhibitors have entered clinical trials. Interestingly, HDAC6 has been shown to affect the acetylation status of heat shock protein 90 (HSP90), which is a key androgen receptor chaperone [12]. HDAC6 inhibitor can decrease AR protein level through dissociating HSP90-AR complex [13, 14].

In this study, we describe the discovery and preclinical development of Zeta55, a dual inhibitor of AR and HADC6 by integrating HDACi zinc-binding group into the optimized skeleton of clinical AR antagonist. Mechanistic studies reveal that Zeta55 not only inhibits AR and HDAC6 activities, but also induces AR degradation. In addition, our work shows that Zeta55 potently inhibits the proliferation of prostate cancer cells *in vitro* and *in vivo*. These data support future clinical development of Zeta55 for the treatment of prostate cancer.

## RESULTS

### Design and docking of Zeta55

**Figure 1:**
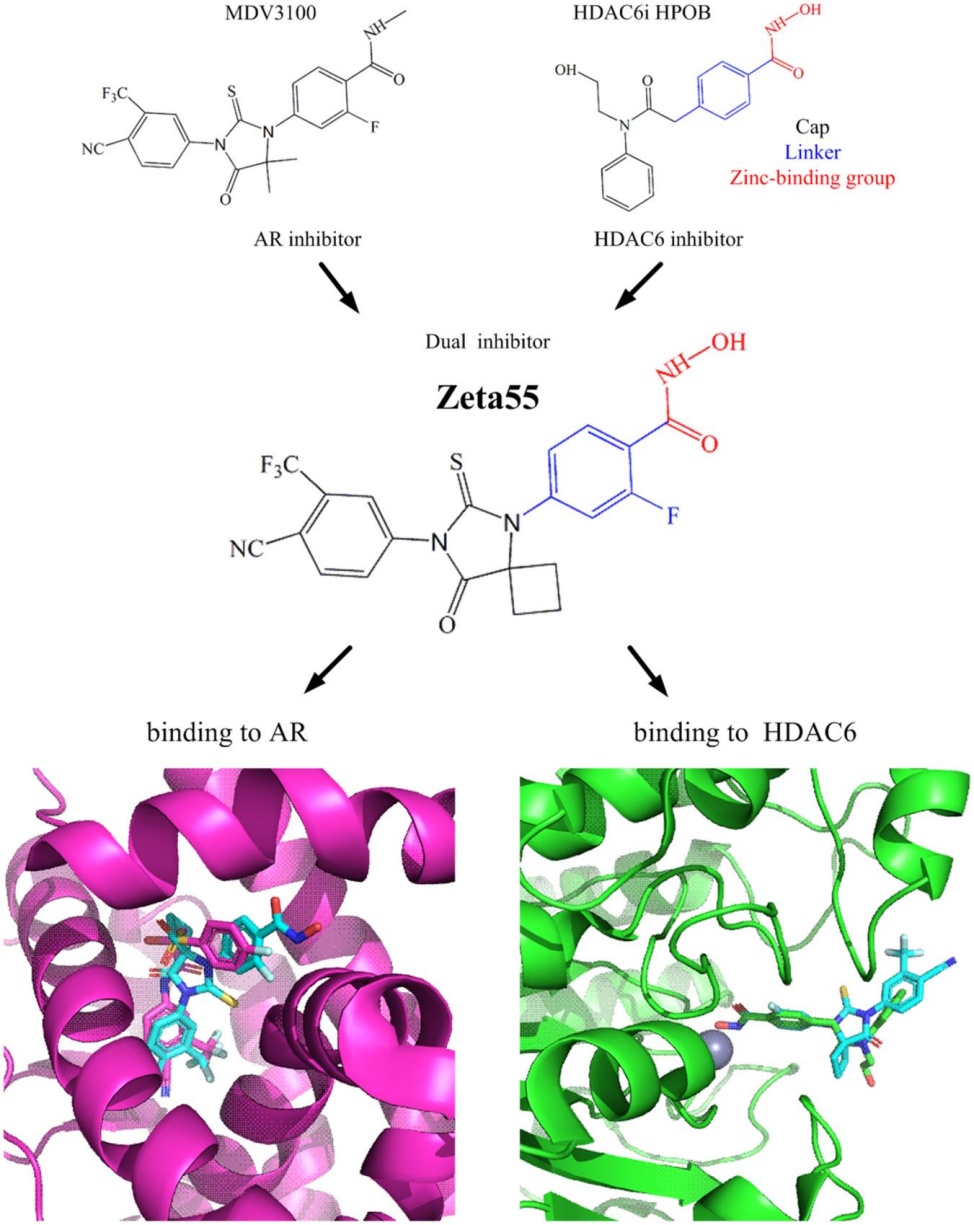
**Design of Zeta55 as a dual inhibitor against AR and HDAC6.** Top left, chemical structures of MDV310; top right, chemical structures of HDAC6 inhibitor HPOB; middle, chemical structure of Zeta55; bottom left, docking of AR with Zeta55 and Bicalutamide; bottom right, docking of HDAC6 with Zeta55 and HPOB. Proteins were presented in cartoon and small molecules were presented in stick. AR/Bicalutamide were colored in mega, Zeta55 was colored in cyan, HDAC6/HPOB were colored in green.

We designed a dual-target inhibitor, Zeta55, which integrates AR and HDAC6 inhibitory functional groups into one single small molecule (molecular weight, 478; Figure 1). We substituted a methyl group in N-methylbenzamide moiety of lutamides with a hydroxyl group. We anticipated that Zeta55 would retain its ability to compete with androgen binding as an AR antagonist, as well as fit well into the substrate-binding pocket of HDAC6. The binding modes of Zeta55 to AR and HDAC6 were predicted using molecular docking. Zeta55 fits well into the ligand binding pocket of AR, and interacts with AR in a manner similar to Bicalutamide in the AR/Bicalutamide structure [15]. In the predicted HDAC6/Zeta55 structure, Zeta55 inserts into HDAC6 catalytic domain with its hydroxamate group, in a manner similar to HPOB binding in the HDAC6/HPOB crystal structure [16].

### Synthesis of Zeta55

**Figure 2:**
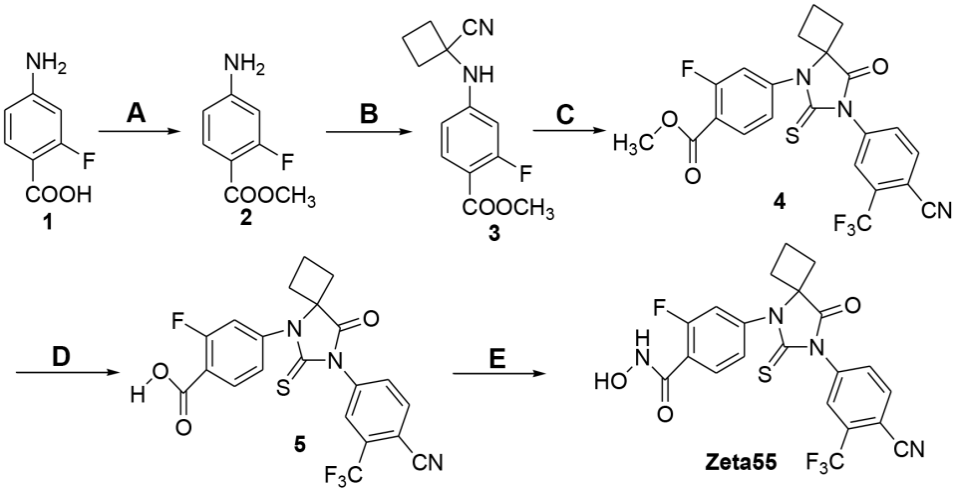
**Synthesis of compound Zeta55.** (**A**) MeOH, SOCl_2_, reflux, 3h, 95.5%; (**B**) cyclobutanone, KCN, HOAc, 80° C, 12h, 92.5%; (**C**) 4-cyano-3-trifluomethylbenzamine, thiophosgene, DMF, 70° C, 24h, 30.2%; (**D**) 1mol/L NaOH, MeOH, 20° C, 4h, 98.2%; (**E**) CDI, NH_2_OH^.^HCl, dry THF, 10h, 28.1%.

Zeta55 was synthesized according to Figure 2. Treatment of 2-fluoro-4-aminobenzoic acid (compound 1) with SOCl_2_ in methanol afforded methyl 2-fluoro-4-aminobenzonate (compound 2), which was reacted with cyclobutanone and KCN via Strecker reaction to generate the cyanoamine (compound 3). The cyclization of cyanoamine with 4-cyano-3-trifluomethylbenzamine in the presence of thiophosgene to give the desired thiohydantoin (compound 4) [17], which was subsequently hydrolyzed with sodium hydroxide to yield compound 5. Finally activation of carboxylic acid with N, N-carbonyldiimidazole followed by the reaction with hydroxylamine hydrochloride yielded the target hydroxamic acid Zeta55 [18].

### Zeta55 inhibits AR and selectively inhibits HDAC6

To evaluate the AR inhibition activities of Zeta55, an AR luciferase activity assay was performed (Table 1 and Supplementary Figure 1). Zeta55 inhibits AR activity with an IC_50_ value of 0.63 μM, which is slightly less potent than MDV3100 (IC_50_: 0.42 μM), suggesting that the substitution of methyl group with hydroxyl group could slightly decrease the binding affinity to AR. An AR competitive binding experiment using fluorescence polarization also reached the same conclusion (Supplementary Figure 2). To evaluate the HDAC inhibition activities of Zeta55, fluorescent-based HDAC assays were performed (Table 1 and Supplementary Figure 3). As a pan-HDAC inhibitor, SAHA potently inhibited the activity of HDAC1, 2, 3, 6. Zeta55 selectively inhibits HDAC6 activity with an IC_50_ value of 0.98 μM (> 20 folds over other HDACs tested). Both compounds seem ineffective against HDAC4. These results suggest that Zeta55 is a HDAC6-selective HDAC inhibitor.

**Table 1:** IC_50_ values of Zeta55 and SAHA against AR and HDACs activities.

**compound**	**IC50 values (μM)**					
	**AR**	**HDAC1**	**HDAC2**	**HDAC3**	**HDAC4**	**HDAC6**
MDV3100	0.42	-	-	-	--	-
SAHA	-	0.12	0.45	0.28	>20	0.16
Zeta55	0.63	>20	>20	>20	>20	0.98

- Not tested.

### Zeta55 inhibits AR-mediated gene transcription

**Figure 3:**
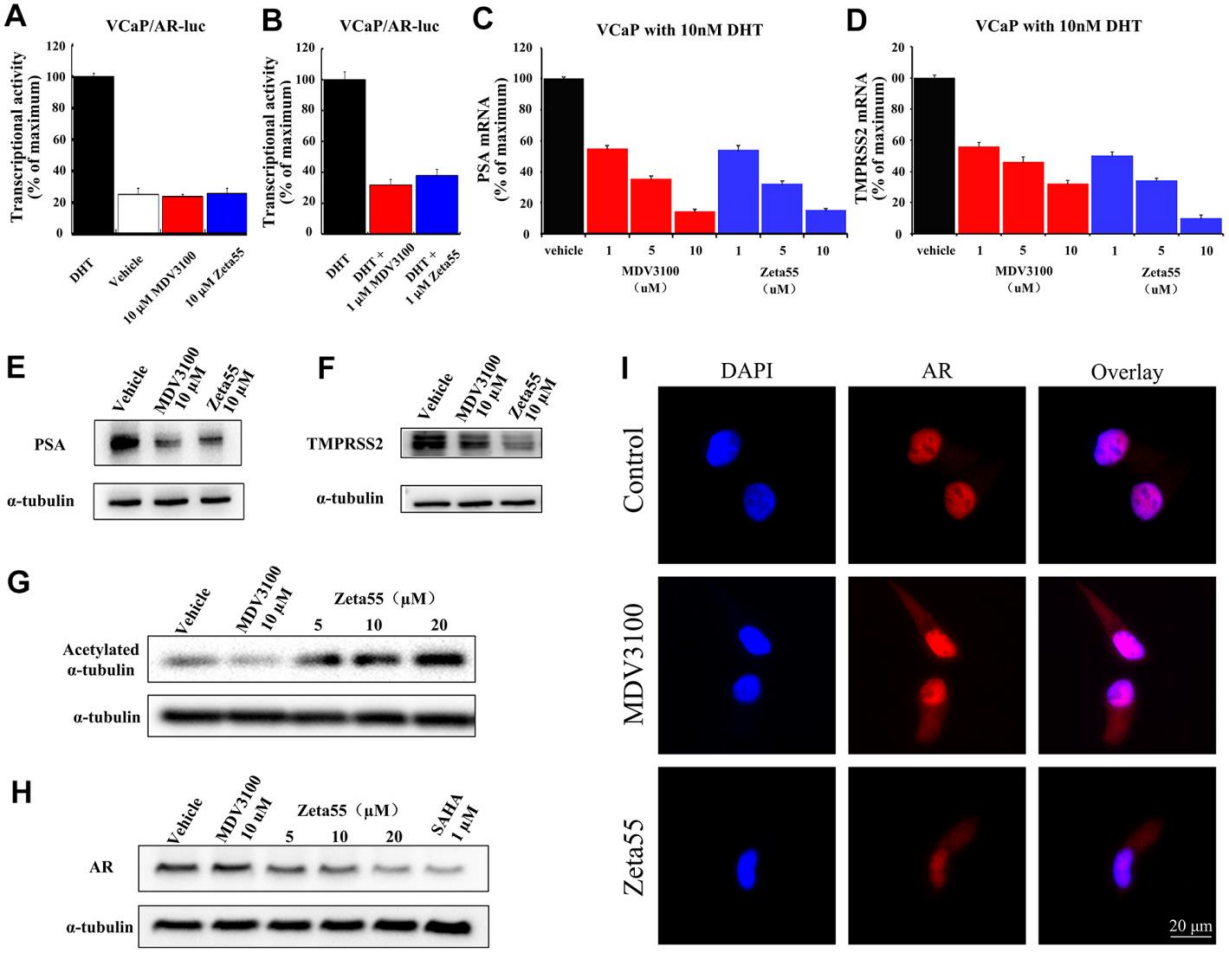
**The AR and HDAC6 inhibitory activity of Zeta55 in cells.** The agonistic effects (**A**) and antagonist effects (**B**) of Zeta55 on AR by reporter gene assay. The VCaP cells were transfected with MMTV-LUC for 24 hours, and then treated with Zeta55 or MDV3100 in the absence of DHT for agonistic mode or with DHT for antagonist mode. Zeta55 decreases RNA expression level of PSA (**C**) and TMPRSS2 (**D**) in VCaP cells. Zeta55 also decreases and protein expression level of PSA (**E**) and TMPRSS2 (**F**) in VCaP cells. The VCaP cells were treated with Zeta55 or MDV3100 together with 10 nM DHT for 24 hours. RNA expression level was analyzed by qRT-PCR and protein expression level was analyzed by Western blot. Zeta55 increases acetylation of α-tubulin (**G**) and decreases AR (**H**) in VCaP cells. VCaP cells were treated with Zeta55, MDV3100 or SAHA for 24 hours before Western blot analysis. (**I**) Representative immunofluorescence microscopic images of VCaP cells for checking the subcellular localization of AR. VCaP cells were cultured with 10% FBS and treated with vehicle (control group),10 μM MDV3100 or Zeta55 for 24 hours, and then stained with DAPI (4’,6-diamidine-2’-phenylindole) and AR.

We further assessed both agonist and antagonist activity of AR signaling in AR-dependent VCaP prostate cancer cell line. Without DHT, 10 μM Zeta55 did not induce the expression of reporter gene, indicating that Zeta55 does not have AR agonist activity (Figure 3A). In the presence of DHT, Zeta55 potently inhibited the AR-mediated expression of reporter gene (Figure 3B).

We then assessed the effects of Zeta55 on the expression of Prostate-specific antigen (PSA) and transmembrane protease serine 2 (TMPRSS2), two endogenous AR-regulated gene. We evaluated both mRNA and protein levels of PSA and TMPRSS2 in VCaP cells. Similar to MDV3100, Zeta55 significantly suppressed DHT-induced endogenous mRNA expression and protein expression of PSA and TMPRSS2 (Figure 3C–3F).

### Zeta55 inhibits HDAC6 activity and decreases AR protein level in VCaP cells

To evaluate the HDAC6 inhibition ability of Zeta55 in VCaP cells, the acetylation status of α-tubulin protein (a HDAC6 substrate) was assessed using western blot. Zeta55 enhanced α-tubulin acetylation in a dose-dependent manner, while MDV3100 did not (Figure 3G). HDAC6 inhibitor has been reported to enhance HSP90 acetylation, and disrupt HSP90-AR interaction, leading to AR degradation [13, 14]. We assessed the protein level of AR in VCaP cells. As shown in Figure 3H, Zeta55 decreased AR protein level in a dose-dependent manner, while MDV3100 did not. Further research found that Zeta555 did not influence expression levels of HSP90 and HSP60, and slightly up-regulated expression levels of HSP70 (Supplementary Figure 4).

### Zeta55 inhibits nuclear translocation of AR

An immunofluorescence experiment of AR and DAPI (4’,6-diamidine-2’-phenylindole) was performed to study the effects of Zeta55 on the localization of AR in VCaP cells. As shown in the Figure 3I, AR of the control group is mainly localized in the nucleus, while AR of the MDV3100 or Zeta55 group distributed in both cytoplasm and nucleus, indicating that Zeta55 can inhibit nuclear translocation of AR.

### Zeta55 inhibits prostate cancer cell proliferation

To evaluate the proliferation inhibitory activity of Zeta55, proliferation assays of VCaP and LNCaP cells (AR overexpressed prostate cancer cell line), DU145 cells (AR negative prostate cancer cell line) and HEK293 cells (embryonic kidney immortalized cell line) were performed using SAHA and MDV3100 as controls (Table 2 and Supplementary Figure 5). Zeta55 inhibited AR-positive VCaP cell proliferation with an IC_50_ value of 2.47 μM, and the inhibition ability was stronger than MDV3100 (IC_50_: 11.04 μM) and SAHA (IC_50_: 4.10 μM). Only SAHA potently inhibited the cell proliferation of AR-negative DU145 cells (IC_50_: 4.02 μM) and HEK293 cells (IC_50_: 4.43 μM). IC_50_ value of Zeta55 on LNCaP cells is higher than that on VCaP cells, which may due to the lower expression of AR in LNCaP cells (Supplementary Figure 6). These data suggest that Zeta55 specifically inhibits the cell proliferation of AR-positive cells, with its inhibitory ability stronger than MDV3100.

**Table 2:** IC_50_ values of Zeta55, MDV3100 and SAHA on the proliferation of VCaP, LNCaP, DU145 and HEK293 cells.

**compound**	**IC50 values (μM)**			
	**VCaP**	**LNCaP**	**DU145**	**HEK293**
SAHA	4.10	3.96	4.02	4.43
MDV3100	11.04	25.81	>50	>50
Zeta55	2.47	8.36	45.67	>50

### Zeta55 potently inhibits tumor growth in a CRPC xenograft model

**Figure 4:**
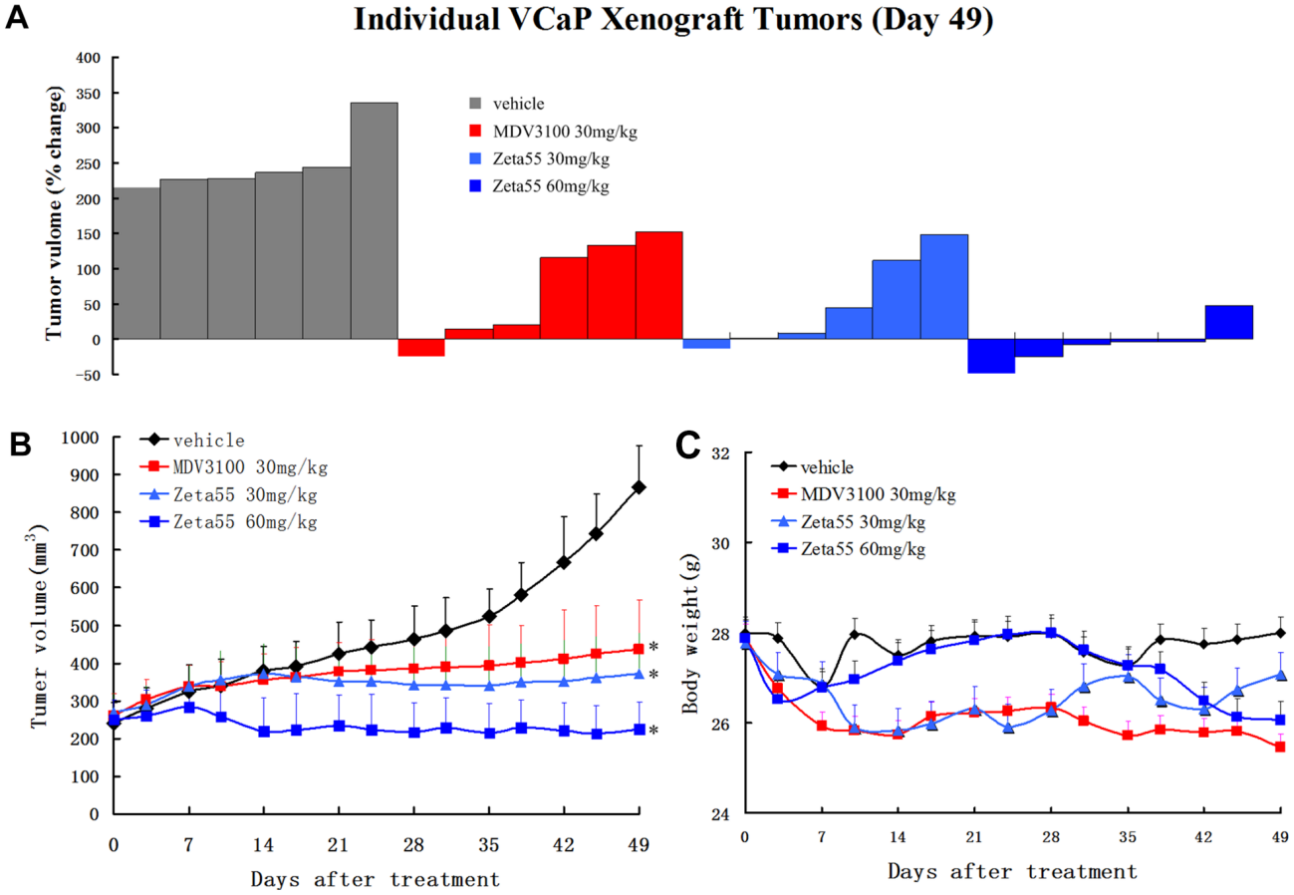
**Zeta55 inhibits tumor growth in a CRPC xenograft model.** (**A**) Percentage change in individual tumor volume of NOD-SCID mice with subcutaneous tumor xenograft grown from VCaP cells. NOD-SCID mice were castrated when the tumor volumes reached about 250mm^3^ on 63 days after subcutaneous injection of VCaP cells. After 21 days when the xenografts continued to grow, the castrated mice were treated with daily oral Zeta55 (30 mg/kg or 60 mg/kg) or MDV3100 (30 mg/kg) for 49 days (n=6). Tumor sizes were monitored twice a week after treatment. (**B**) Mean tumor volumes (mm^3^, +SEM) of NOD-SCID mice after treatment. (**C**) Mean body weight (g, +SEM) of NOD-SCID mice after treatment. *, P < 0.01 vs. vehicle.

To evaluate the *in vivo* pharmacodynamic activity of Zeta55, castrated male immunodeficient mice harboring VCaP xenograft tumors were orally treated with vehicle, MDV3100 (30 mg/kg, daily), Zeta55 (30 mg/kg, daily) or Zeta55 (60 mg/kg, daily). Zeta55 (30 mg/kg/d) showed slightly better antitumor activity than MDV3100 (30 mg/kg/d). A higher Zeta55 dose (60 mg/kg/d) led to increased efficacy (5 of 6 tumors with tumor regression) (Figure 4A, 4B). During the treatment, the weights of mice treated with Zeta55 at both doses did not decrease significantly (Figure 4C).

**Figure 5:**
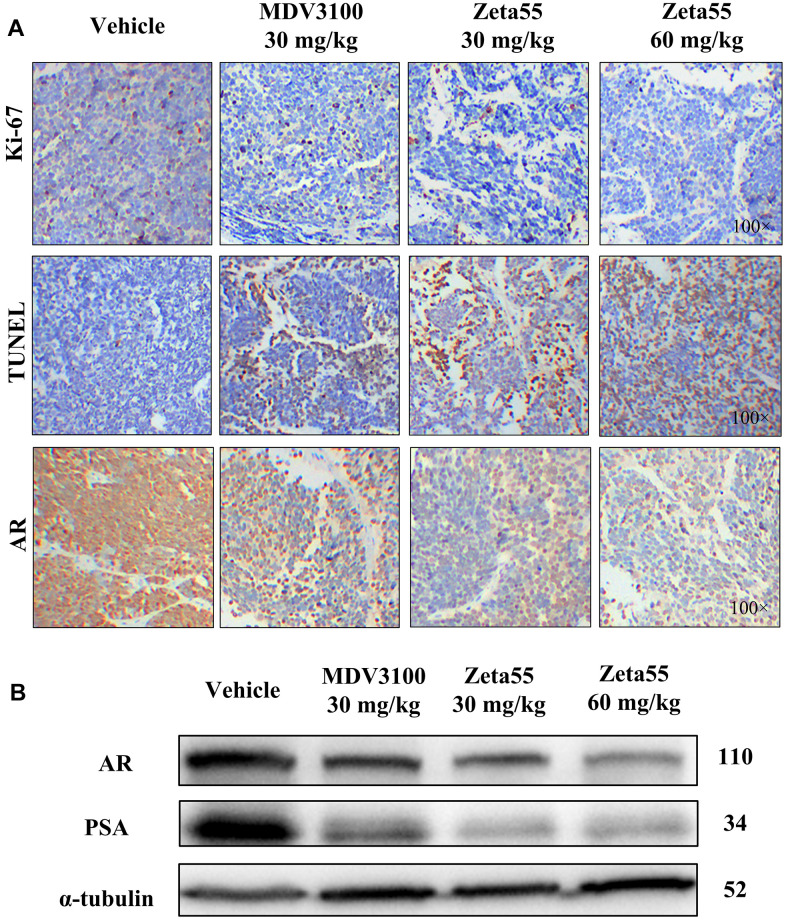
**Immunohistochemistry and Western blot analysis of tumor xenografts.** (**A**) Ki67, TUNEL and AR of different group xenografts were stained and evaluated at 100× magnifications. (**B**) AR and PSA of a representative xenograft in each group were analyzed by Western blot.

Ki-67 and TUNEL staining of tumor xenografts showed that Zeta55 inhibited tumor cell proliferation and induced apoptosis (Figure 5A). Immunohistochemical and Western blot analysis of Zeta55-treated tumors showed decreased expression of AR and PSA compared to MDV3100 and vehicle (Figure 5A, 5B). These results are consistent with our findings *in vitro*.

### Pharmacokinetics of Zeta55 in rats

To evaluate the pharmacokinetic properties of Zeta55 *in vivo*, rats were orally (20 mg/kg) or intravenously (5 mg/kg) administrated with Zeta55, and the plasma concentration of Zeta55 was measured using LC-MS/MS. The key pharmacokinetic parameters of Zeta55 are summarized in Table 3. The half-lives (*t*_1/2_) of Zeta55 in rats were about 0.8 hour for intravenous administration, and 2.9 hours for oral administration. The oral bioavailability of Zeta55 in rats was 17.6%.

**Table 3:** Pharmacokinetic parameters of Zeta55 in rats.

	**t**_1/2_ **(hr)**	**T**_max_ **(hr)**	**C**_max_ **(ng/mL)**	**AUC**_0-t_ (hr*ng/mL)	**AUC**_0-∞_ (hr*ng/mL)	**Vz/F** **(mL/kg)**	**Cl/F** **(mL/hr/kg)**	**Bioavailability** **(%)**
PO	2.925	0.375	182.725	508.153	555.388	166146.93	37915.801	17.60 %
IV	0.780	0.083	1434	721.945	727.072	7741.576	6876.894	-

PO, Oral gavage (20 mg / kg, n=4); IV, Tail vein injection (5 mg/kg, n=1); t_1/2_, Terminal half-life in plasma; T_max_, Time to Maximum Plasma; Concentration; C_max_, Maximal concentration in plasma; AUC_0-t_, Area under the plasma concentration curve from time 0-72h; AUC_0-∞_, Area under the plasma concentration curve from time 0-infinity; Vz/F, apparent volume of distribution, Cl/F, Clearance rate.

## DISCUSSION

The combination therapy that two or more drugs are used in combination to treat disease attract increasing attention in new drug discovery and development [19]. In prostate cancer, one combination therapy of targeted alpha therapy (radium-223) with AR antagonist (MDV3100) or immunotherapies (pembrolizumab or atezolizumab) is now in phase I/II clinical trials [20, 21]; while other combination therapies of AR antagonist (MDV3100 or ARN-509) with CYP17, PARP or VEGFR inhibitors are also in clinical trials [22]. In this study, we show that Zeta55 can target both AR and HDAC6, and potently inhibit the tumor growth of prostate cancer *in vitro* and *in vivo*. Compared to single-target drugs, this multiple-target drug Zeta55 exhibits stronger anti-proliferation activity than MDV3100 and SAHA in VCaP cell experiments and better antitumor activity than MDV3100 in CRPC xenograft model. Compared to the combination of existing drugs in prostate cancer, this multiple-target drug Zeta55 may provide higher therapeutic effects and fewer side effects, as well as more predictable pharmacokinetic profiles and better patient compliance [23, 24].

Other multiple-target drugs have also been developed for treating prostate cancers. For example, CFG920, a dual inhibitor of CYP17 and CYP11B2, is in clinical phase II trials [25, 26]. Galeterone, a potent dual inhibitor of CYP17 and AR, is more effective than castration for treating prostate cancer in tumor xenograft model and is also effective in phase I/II clinical studies [27–29]. A series of steroidal imidazoles designed as dual inhibitors of CYP17 and AR were reported recently and the most potent compound of them suppressed LNCaP cell proliferation more effectively than Flutamide [30]. Similar to our study, previous literature has reported two class hybrid molecules of AR antagonist and pan-HDACi for treating prostate cancer [31, 32]. Those compounds gain their HDAC inhibitor activity by adding a hydroxamate group with a linker; while in this study, Zeta55 gains its HDAC6 inhibitor activity only by substituting a methyl group in N-methylbenzamide moiety of lutamides with a hydroxyl group. Compared to those compounds, Zeta55 has the following advantages. First, Zeta55 (molecular weight: 478) has a smaller molecular weight. Second, Zeta55, with its six rotatable bonds, has fewer rotatable bonds. Third, Zeta55 is more selective for HDAC6. Based on these characteristics, Zeta55 might have more favorable drug-like properties than those compounds described in the previous literatures.

Coincident with the design strategy, Zeta55 inhibits both AR and HDAC6 activities in both *in vitro* and *in vivo* functional assays. The AR binding activity of Zeta55 was slightly weaker than MDV3100, indicating that substituting a methyl group with a hydroxyl group affects the binding affinity to AR. Meanwhile, Zeta55 has less potent HDAC6 inhibition than SAHA, with an IC_50_ of 0.98 μM. However, combining these two suboptimal activities appears to have a synergistic effect on inhibiting the cell proliferation in prostate cancer. In the cell proliferation assays of VCaP cells, with 60% AR binding affinity of MDV3100, Zeta55 exhibits 4.5-fold stronger anti-proliferation activity than MDV3100.

**Figure 6:**
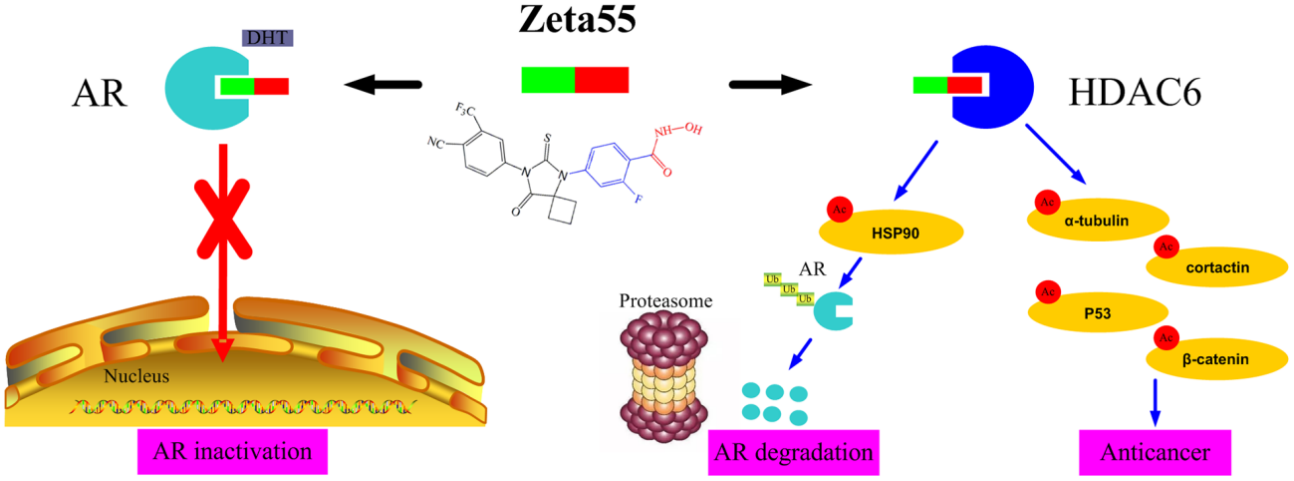
**The anti-cancer mechanisms of Zeta55 in prostate cancer.** First, Zeta55 prevents androgens from binding AR, leading to AR inactivation. Second, Zeta55 inhibits the deacetylation activity of HDAC6, which regulates cell proliferation, metastasis, invasion, and mitosis. Third, Zeta55 promotes AR degradation through its HDAC6 inhibitor activity.

The potent anti-proliferation activity of Zeta55 could be attributed to its unique combination of multiple mechanisms (Figure 6). First, like the first and second generations of AR antagonists, Zeta55 prevents androgens from binding AR, leading to AR inactivation. Second, Zeta55 inhibits the deacetylation activity of HDAC6, which regulates cell proliferation, metastasis, invasion, and mitosis in tumors [11]. Third, Zeta55 promotes AR degradation through its HDAC6 inhibitory activity. This is especially important because AR protein overexpression is commonly associated with the development of CRPC.

Previous study reported that MDV3100 has an oral bioavailability of 97% and a plasma half-life of 8.56 hours in rats [33]. Our pharmacokinetic analyses show that the oral bioavailability of Zeta55 in rats is 17.6% with a plasma half-life of 2.9 hours. These results suggest that the systemic exposure of MDV3100 in rodents is much higher than that of Zeta55. Nevertheless, Zeta55 showed slightly better anti-tumor activity than MDV3100 at 30 mg/kg/d. A higher dose of Zeta55 (60 mg/kg/d) showed a much better anti-tumor activity of MDV3100 (30 mg/kg/d). It's worth noting that MDV3100 has a long plasma half-life in humans (5.8 days), which may lead to drug accumulation in human body [34]. It is possible that Zeta55 may have better pharmacokinetic properties in humans than MDV3100.

In summary, our findings show that Zeta55 is more efficacious than MDV3100 in nonclinical *in vitro* and *in vivo* studies of CRPC. Zeta55, with its unique and novel multitarget activities, is suitable for future clinical development in CRPC treatment.

## MATERIALS AND METHODS

### Compounds and cell lines

Zeta55 and MDV3100 were synthesized in laboratory. SAHA was purchased from ApexBio Technology, and dihydrotestosterone (DHT) was purchased from Sigma-Aldrich. All compounds were dissolved in dimethyl sulfoxide (DMSO) for *in vitro* assays. For *in vivo* studies in the mouse model, Zeta55 and MDV3100 were dissolved in a formulation of 1% carboxymethyl cellulose, 0.1% Tween-80 and 5% DMSO.

VCaP, LNCaP, DU145 and HEK293 cells were obtained from the Cell Bank of the Chinese Academy of Sciences (Shanghai, China) with STR Authentication. For subculturing, VCaP cells were cultured in Dulbecco's modified Eagle's medium (DMEM) with 10% FBS and digested by phenol-red-free TrypLE Express (Gibco). For AR antagonist or agonist assays, VCaP cells were cultured in steroid-free assay medium with or without 10 nM DHT. The steroid-free assay medium consisted of phenol-red-free DMEM (Gibco) and 5% charcoal-stripped fetal bovine serum (HyClone). LNCaP, DU145 and HEK293 cells were cultured in DMEM with 10% FBS.

### Molecular docking

Computational docking was performed to predict the binding of Zeta55 to AR and HDAC6. The chemical structure of Zeta55 was drawn using ChemDraw and the 3D coordinate was generated in PRODRG [35]. AutoDock Tools 1.5.6 [36] was then used to assign hydrogens, Gasteiger charges and rotatable bonds to the compounds. The PDB accession numbers of the structure models used for docking are 1Z95 (AR) and 5EF7 (HDAC6). A docking grid with the dimensions of 40*40*40 Å3, encompassing the entire ligand-binding clefts, was used.

### Reporter gene assays

VCaP cells were seeded in 96-well plates and cultured in steroid-free assay medium for 24 hours. After adherence, VCaP cells were transfected with MMTV-LUC using Lipofectamine 3000 (Invitrogen) for 24 hours and then treated with Zeta55 or MDV3100 together with 10 nM DHT for AR antagonist assays or without DHT for AR agonist assays. After 48 hours, the cell extracts were tested for luciferase activity using a Steady-Lumi™ Firefly Luciferase Assay Kit (Beyotime Biotechnology).

### HDAC assay

Commercial fluorescent assay kits were used to measure the activities of HDAC (BPS Bioscience). Briefly, the tested compounds were diluted in DMSO into a 96-well plate, and then assay buffer, HDAC, and substrate were added. The plate was incubated at 37° C for 30 minutes for enzymatic reactions, and the developer was added. After 10 minutes of incubation, the generated fluorescence was measured at an excitation of 380 nm and an emission of 460 nm.

### Cell proliferation assay

To measure AR-mediated cell proliferation, 10,000 VCaP cells (AR positive prostate cancer cells) were plated in each well of a 96-well plate and incubated in steroid-free assay medium for 12 hours. After adherence, VCaP cells were treated with increasing concentrations of Zeta55, MDV3100 or SAHA together with 10 nM DHT for AR antagonist assays or without DHT for AR agonist assays. After 7 days, cell viability was measured using a CellTiter 96® AQueous One Solution Cell Proliferation Assay kit (Promega). 8,000 LNCaP cells, 5,000 DU145 cells (AR negative prostate cancer cells) or HEK293 cells (AR negative noncancerous cells) per well were cultured in DMEM with 10% FBS for 12 hours. After treatment with increasing concentrations of Zeta55, MDV3100 or SAHA for 3 days, the cell viability was measured using a CellTiter 96® AQueous One Solution Cell Proliferation Assay kit (Promega).

### qRT-PCR

The RNA of VCaP cells was extracted using a Simply P Total RNA Extraction kit (Bioer technology) and cDNA was synthesized using a SMARTer PCR cDNA Synthesis Kit (Clontech). PSA and β-actin primers were synthesized by Sangon Biotech according to previous literature [37]. Real-time PCR was performed using the PowerUp SYBR™ Green kit (ThermoFisher). The PCR program was run on a LightCycler 480II (Roche) and set as follows: 2 minutes at 95° C followed by 40 cycles of 30 seconds at 95° C, 15 seconds at 60° C and 30 seconds at 72° C. Data were quantified using the 2-ΔΔCt method based on the Ct values of PSA and β-actin from two parallel experiments performed in triplicate.

### Western blot analysis

The whole-cell extracts were prepared in lysis buffer with a protease inhibitor cocktail (Bimake), while the xenograft tissue extracts were prepared in 0.5% Nonidet P 40 Substitute solution (Biosharp) with cocktail protease inhibitor (Bimake). Each sample containing 30 μg of total protein was loaded onto 10% SDS-PAGE and electrotransferred onto polyvinylidene fluoride membranes. The membranes were blocked with 5% skimmed milk in PBST at room temperature for 2 hours and incubated with primary antibodies at 4° C overnight. After the membranes were washed with PBST three times, they were incubated with horseradish peroxidase-conjugated anti-rabbit or anti-mouse secondary antibody at room temperature for 1 hour. Chemiluminescent detection was performed with WesternBright ECL (Advansta). The immunoblots were visualized using the FluorChem FC3 system (ProteinSimple). Antibodies: AR (SANTA, sc-7305), PSA (SANTA, sc-7316), acetylated α-tubulin (CST, 5335s), and α-tubulin (Proteintech, 66031-1-lg).

### Immunofluorescence

VCaP cells (1×10^6^ cells/well) were seeded on coverslips in 6-well plates and treated by Zeta55 (10 μM), MDV3100 (10μM) or DMSO for 24 hours. Cells were fixed with 4% paraformaldehyde for 15 minutes at room temperature, permeabilized with immunostaining permeate for 10 minutes and blocked with 5% BSA containing 1% Tween-20 for 1 hour. After overnight Immunostain with AR Rabbit monoclonal antibody (1:400;CST,5153S) at 4° C, a Cy3 conjugated Goat Anti-Rabbit IgG (1:300; Servicebio, GB21303) reacted for 1 hour. DAPI (4’,6-diamidine-2’-phenylindole, Servicebio, G1012) stained the nuclear to bule for 10 minutes in the dark. Washing three times with PBS between each step. Immunofluorescence images were acquired by fluorescence microscopy using a NIKON ECLIPSE CI microscope (Nikon Corporation; magnification).

### The castration-resistant VCaP xenograft mouse model

The animal protocols used in this study were evaluated and approved by the Xiangya Hospital Medical ethics committee of Central South University. NOD-SCID mice were purchased from Hunan SJA Laboratory Animal Co. Ltd. with a quality license and raised in the SPF Laboratory Animal Room of the Department of Laboratory Zoology at Central South University. Briefly, VCaP cells were suspended in DMEM and Matrigel (BD) (1:1), and 5×10^6^ cells/100 μl were subcutaneously injected into a 7-week-old male NOD-SCID mouse. After the average tumor volume reached approximately 250 mm^3^ (63 days after injection), all NOD-SCID mice were castrated under anesthesia. When the xenografts resumed growth after 21 days of recovery, the castrated mice were randomly divided into four groups (n=6) and were orally treated with vehicle, MDV3100 (30 mg/kg, daily) or Zeta55 (30 or 60 mg/kg, daily) for 49 days. VCaP xenografts were measured twice per week in two dimensions (L and W) by a Vernier caliper, and the tumor volume was calculated as W^2^×L/2 (mm^3^).

### Immunohistochemistry

Formalin-fixed, paraffin-embedded xenograft tissue sections were used for immunohistochemistry. TUNEL staining was performed according to the instructions in the TUNEL kit (KeyGEN). Tissue sections were deparaffinized and then boiled in 0.01 mol/L sodium citrate buffer (pH 6.0) in a kilowatt microwave oven for 10 minutes to retrieve cell antigens. Ki67 (Abcam, ab15580) or AR (SANTA, sc-7305) was used as the primary antibody. All tissue sections were immunohistochemically stained using the avidin-biotin-peroxidase method and were counterstained with hematoxylin. The staining intensities were evaluated at 100× magnification.

### Pharmacokinetics

SD male rats weighing about 250 g were used for the pharmacokinetics study of Zeta55. After a fast of 12 hours, four rats were oral administrated with 20 mg/kg Zeta55 and one rat was tail vein injected with 5 mg/kg Zeta55. Plasma samples of 0.2 ml were taken at 0, 0.083, 0.25, 0.5, 1, 2, 3, 5, 8, 10, 24, 48, and 72 hours after administration and then measured by LC-MS/MS. AUC_0-t_, AUC_0-∞_, t_1/2,_ T_max_, Vz/F, and Cl/F were analyzed by Phoenix winnonlin 8.0.

## Supplementary Material

Supplementary Figures

## References

[1] Siegel RL, Miller KD, Jemal A. Cancer statistics, 2019. CA Cancer J Clin. 2019; 69:7–34. 10.3322/caac.21551 30620402

[2] Tan MH, Li J, Xu HE, Melcher K, Yong EL. Androgen receptor: structure, role in prostate cancer and drug discovery. Acta Pharmacol Sin. 2015; 36:3–23. 10.1038/aps.2014.18 24909511 PMC4571323

[3] Crona DJ, Milowsky MI, Whang YE. Androgen receptor targeting drugs in castration-resistant prostate cancer and mechanisms of resistance. Clin Pharmacol Ther. 2015; 98:582–89. 10.1002/cpt.256 26331358 PMC4715745

[4] Nevedomskaya E, Baumgart SJ, Haendler B. Recent advances in prostate cancer treatment and drug discovery. Int J Mol Sci. 2018; 19:1359. 10.3390/ijms19051359 29734647 PMC5983695

[5] Alghazo O, Thangasamy I, Sathianathen N, Murphy DG. Re: darolutamide in nonmetastatic castration-resistant prostate cancer. Eur Urol. 2019; 76:536–37. 10.1016/j.eururo.2019.04.028 31101535

[6] Mateo J, Smith A, Ong M, de Bono JS. Novel drugs targeting the androgen receptor pathway in prostate cancer. Cancer Metastasis Rev. 2014; 33:567–79. 10.1007/s10555-013-9472-2 24390422

[7] Ganai SA. Histone deacetylase inhibitor sulforaphane: the phytochemical with vibrant activity against prostate cancer. Biomed Pharmacother. 2016; 81:250–57. 10.1016/j.biopha.2016.04.022 27261601

[8] Bolden JE, Peart MJ, Johnstone RW. Anticancer activities of histone deacetylase inhibitors. Nat Rev Drug Discov. 2006; 5:769–84. 10.1038/nrd2133 16955068

[9] Wang XX, Wan RZ, Liu ZP. Recent advances in the discovery of potent and selective HDAC6 inhibitors. Eur J Med Chem. 2018; 143:1406–18. 10.1016/j.ejmech.2017.10.040 29133060

[10] Peng C, Yao G, Gao BM, Fan CX, Bian C, Wang J, Cao Y, Wen B, Zhu Y, Ruan Z, Zhao X, You X, Bai J, et al. High-throughput identification of novel conotoxins from the Chinese tubular cone snail (Conus betulinus) by multi-transcriptome sequencing. Gigascience. 2016; 5:17. 10.1186/s13742-016-0122-9 27087938 PMC4832519

[11] Li T, Zhang C, Hassan S, Liu X, Song F, Chen K, Zhang W, Yang J. Histone deacetylase 6 in cancer. J Hematol Oncol. 2018; 11:111. 10.1186/s13045-018-0654-9 30176876 PMC6122547

[12] Ai J, Wang Y, Dar JA, Liu J, Liu L, Nelson JB, Wang Z. HDAC6 regulates androgen receptor hypersensitivity and nuclear localization via modulating Hsp90 acetylation in castration-resistant prostate cancer. Mol Endocrinol. 2009; 23:1963–72. 10.1210/me.2009-0188 19855091 PMC2796151

[13] Chen L, Meng S, Wang H, Bali P, Bai W, Li B, Atadja P, Bhalla KN, Wu J. Chemical ablation of androgen receptor in prostate cancer cells by the histone deacetylase inhibitor LAQ824. Mol Cancer Ther. 2005; 4:1311–19. 10.1158/1535-7163.MCT-04-0287 16170022

[14] Gibbs A, Schwartzman J, Deng V, Alumkal J. Sulforaphane destabilizes the androgen receptor in prostate cancer cells by inactivating histone deacetylase 6. Proc Natl Acad Sci USA. 2009; 106:16663–68. 10.1073/pnas.0908908106 19805354 PMC2757849

[15] Bohl CE, Gao W, Miller DD, Bell CE, Dalton JT. Structural basis for antagonism and resistance of bicalutamide in prostate cancer. Proc Natl Acad Sci USA. 2005; 102:6201–06. 10.1073/pnas.0500381102 15833816 PMC1087923

[16] Hai Y, Christianson DW. Histone deacetylase 6 structure and molecular basis of catalysis and inhibition. Nat Chem Biol. 2016; 12:741–47. 10.1038/nchembio.2134 27454933 PMC4990478

[17] Jung ME, Ouk S, Yoo D, Sawyers CL, Chen C, Tran C, Wongvipat J. Structure-activity relationship for thiohydantoin androgen receptor antagonists for castration-resistant prostate cancer (CRPC). J Med Chem. 2010; 53:2779–96. 10.1021/jm901488g 20218717 PMC3180999

[18] Usachova N, Leitis G, Jirgensons A, Kalvinsh I. Synthesis of Hydroxamic Acids by Activation of Carboxylic Acids with N,N '-Carbonyldiimidazole: Exploring the Efficiency of the Method. Synth Commun. 2010; 40:927–35. 10.1080/00397910903026723

[19] Anighoro A, Bajorath J, Rastelli G. Polypharmacology: challenges and opportunities in drug discovery. J Med Chem. 2014; 57:7874–87. 10.1021/jm5006463 24946140

[20] Parker C, Heidenreich A, Nilsson S, Shore N. Current approaches to incorporation of radium-223 in clinical practice. Prostate Cancer Prostatic Dis. 2018; 21:37–47. 10.1038/s41391-017-0020-y 29298991 PMC5895600

[21] De Vincentis G, Gerritsen W, Gschwend JE, Hacker M, Lewington V, O'Sullivan JM, Oya M, Pacilio M, Parker C, Shore N, Sartor O. Targeted Alpha Therapy Prostate Working Group. Advances in targeted alpha therapy for prostate cancer. Ann Oncol. 2019; 30:1728–39. 10.1093/annonc/mdz270 31418764 PMC6927314

[22] Mostaghel EA, Plymate SR, Montgomery B. Molecular pathways: targeting resistance in the androgen receptor for therapeutic benefit. Clin Cancer Res. 2014; 20:791–98. 10.1158/1078-0432.CCR-12-3601 24305618 PMC3944407

[23] Zhang W, Bai Y, Wang Y, Xiao W. Polypharmacology in drug discovery: a review from systems pharmacology perspective. Curr Pharm Des. 2016; 22:3171–81. 10.2174/1381612822666160224142812 26907941

[24] Bhatia S, Krieger V, Groll M, Osko JD, Reßing N, Ahlert H, Borkhardt A, Kurz T, Christianson DW, Hauer J, Hansen FK. Discovery of the first-in-class dual histone deacetylase-proteasome inhibitor. J Med Chem. 2018; 61:10299–309. 10.1021/acs.jmedchem.8b01487 30365892 PMC6249066

[25] Alex AB, Pal SK, Agarwal N. CYP17 inhibitors in prostate cancer: latest evidence and clinical potential. Ther Adv Med Oncol. 2016; 8:267–75. 10.1177/1758834016642370 27482286 PMC4952018

[26] Gaul C, Mistry P, Moebitz H, Perrone M, Gruenenfelder B, Guerreiro N, Hackl W, Wessels P, Berger E, Bock M, Sengupta S, Rao V, Ramachandra M, et al. Discovery of CFG920, a dual CYP17/CYP11B2 inhibitor, for the treatment of castration resistant prostate cancer. Conference: 250th ACS National Meeting: Boston, USA. Abstr Pap Am Chem S. 2015.

[27] Handratta VD, Vasaitis TS, Njar VC, Gediya LK, Kataria R, Chopra P, Newman D Jr, Farquhar R, Guo Z, Qiu Y, Brodie AM. Novel C-17-heteroaryl steroidal CYP17 inhibitors/antiandrogens: synthesis, *in vitro* biological activity, pharmacokinetics, and antitumor activity in the LAPC4 human prostate cancer xenograft model. J Med Chem. 2005; 48:2972–84. 10.1021/jm040202w 15828836

[28] Bastos DA, Antonarakis ES. Galeterone for the treatment of advanced prostate cancer: the evidence to date. Drug Des Devel Ther. 2016; 10:2289–97. 10.2147/DDDT.S93941 27486306 PMC4956059

[29] McClurg UL, Azizyan M, Dransfield DT, Namdev N, Chit NC, Nakjang S, Robson CN. The novel anti-androgen candidate galeterone targets deubiquitinating enzymes, USP12 and USP46, to control prostate cancer growth and survival. Oncotarget. 2018; 9:24992–5007. 10.18632/oncotarget.25167 29861848 PMC5982776

[30] Hou Q, He C, Lao K, Luo G, You Q, Xiang H. Design and synthesis of novel steroidal imidazoles as dual inhibitors of AR/CYP17 for the treatment of prostate cancer. Steroids. 2019; 150:108384. 10.1016/j.steroids.2019.03.003 30885648

[31] Rosati R, Chen B, Patki M, McFall T, Ou S, Heath E, Ratnam M, Qin Z. Hybrid enzalutamide derivatives with histone deacetylase inhibitor activity decrease heat shock protein 90 and androgen receptor levels and inhibit viability in enzalutamide-resistant C4-2 prostate cancer cells. Mol Pharmacol. 2016; 90:225–37. 10.1124/mol.116.103416 27382012 PMC4998664

[32] Gryder BE, Akbashev MJ, Rood MK, Raftery ED, Meyers WM, Dillard P, Khan S, Oyelere AK. Selectively targeting prostate cancer with antiandrogen equipped histone deacetylase inhibitors. ACS Chem Biol. 2013; 8:2550–60. 10.1021/cb400542w 24004176 PMC3836611

[33] Ohtsu Y, Gibbons JA, Suzuki K, Fitzsimmons ME, Nozawa K, Arai H. Absorption, distribution, metabolism, and excretion of the androgen receptor inhibitor enzalutamide in rats and dogs. Eur J Drug Metab Pharmacokinet. 2017; 42:611–26. 10.1007/s13318-016-0374-x 27590197

[34] Gibbons JA, Ouatas T, Krauwinkel W, Ohtsu Y, van der Walt JS, Beddo V, de Vries M, Mordenti J. Clinical pharmacokinetic studies of enzalutamide. Clin Pharmacokinet. 2015; 54:1043–55. 10.1007/s40262-015-0271-5 25917876 PMC4580721

[35] Schüttelkopf AW, van Aalten DM. PRODRG: a tool for high-throughput crystallography of protein-ligand complexes. Acta Crystallogr D Biol Crystallogr. 2004; 60:1355–63. 10.1107/S0907444904011679 15272157

[36] Morris GM, Huey R, Lindstrom W, Sanner MF, Belew RK, Goodsell DS, Olson AJ. AutoDock4 and AutoDockTools4: automated docking with selective receptor flexibility. J Comput Chem. 2009; 30:2785–91. 10.1002/jcc.21256 19399780 PMC2760638

[37] Clegg NJ, Wongvipat J, Joseph JD, Tran C, Ouk S, Dilhas A, Chen Y, Grillot K, Bischoff ED, Cai L, Aparicio A, Dorow S, Arora V, et al. ARN-509: a novel antiandrogen for prostate cancer treatment. Cancer Res. 2012; 72:1494–503. 10.1158/0008-5472.CAN-11-3948 22266222 PMC3306502

