# Supplementary material for: Discovery of a novel AR/HDAC6 dual inhibitor for prostate cancer treatment
Source: Aging (Albany NY). 2021 Feb 17;13(5):6982–98. doi: 10.18632/aging.202554 (PMC7993727; doi:10.18632/aging.202554)
Supplement: Supplementary Figures [file aging-13-202554-s001.pdf]

SUPPLEMENTARY FIGURES

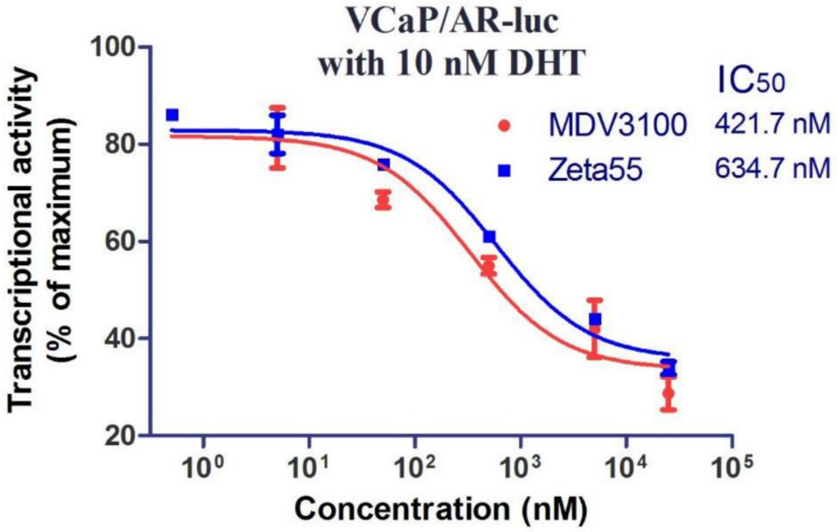

Supplementary Figure 1. The inhibition effects of Zeta55 and MDv3100 on AR transcriptional activity using an AR luciferase assay.

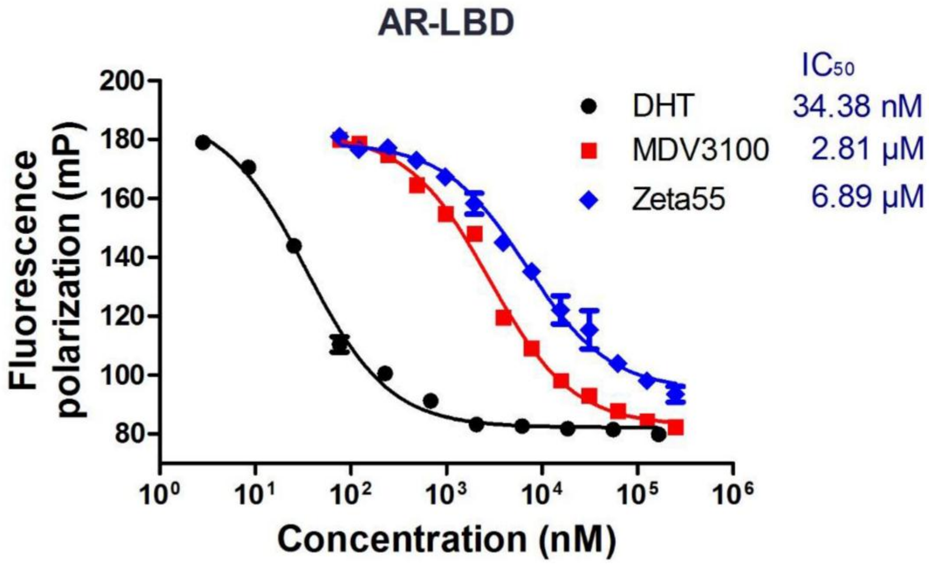

Supplementary Figure 2. The binding affinities of Zeta55, MDV3100 and DHT to AR-LBD were measured by a fluorescence polarization assay. Test compounds were incubated with recombinant AR-LBD (GST) and the fluorescence polarization (mP) was measured at an excitation wavelength of 485 nm and emission wavelength of 535 nm.

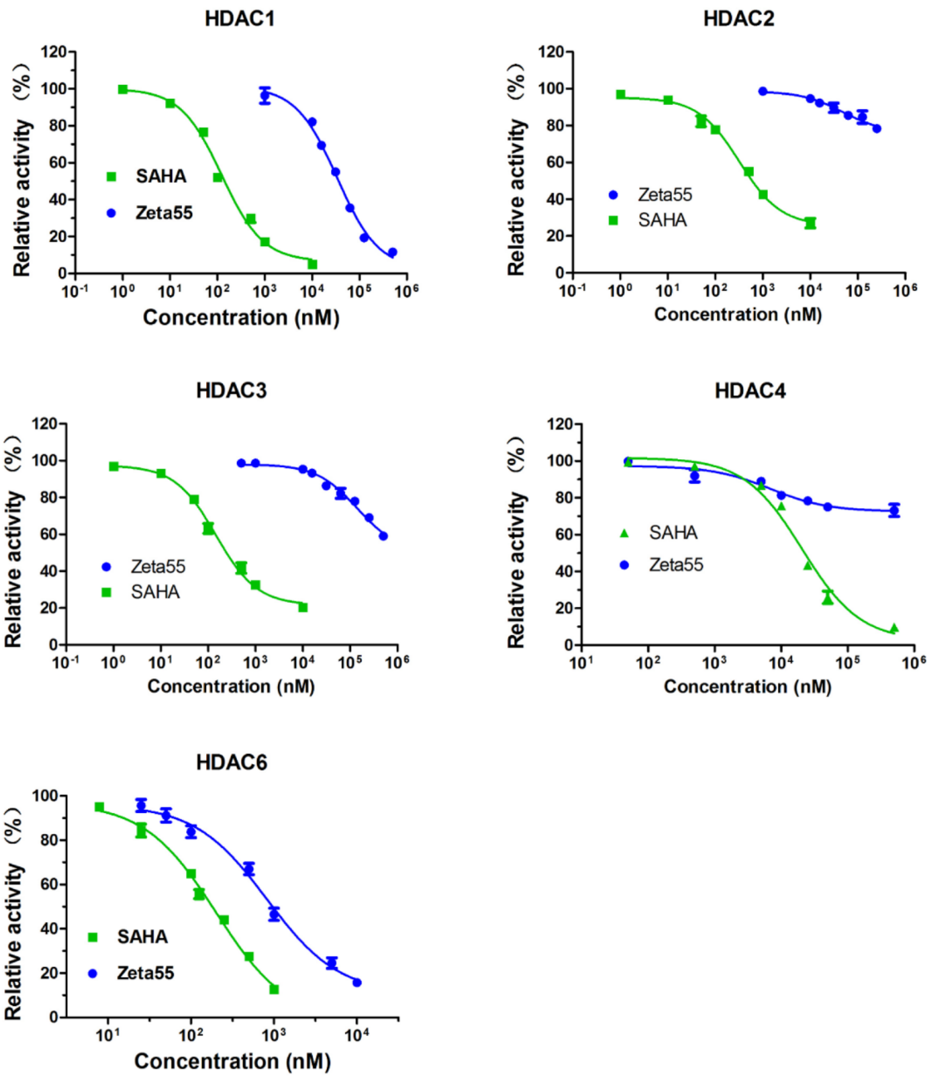

Supplementary Figure 3. The HDAC1, 2, 3, 4 and 6 activities of Zeta55 and SAHA.

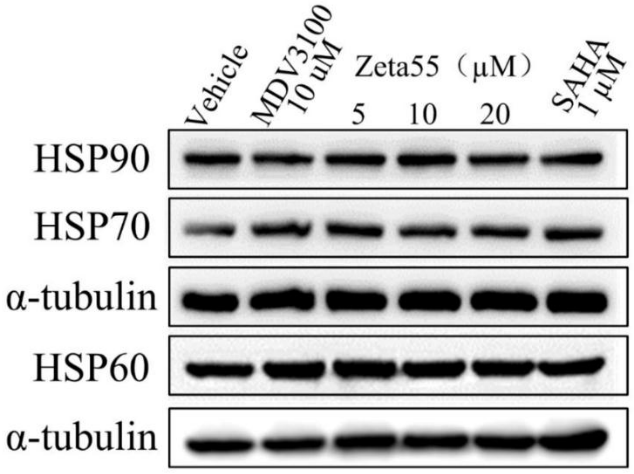

Supplementary Figure 4. Western blot of HSP90, HSP70 and HSP60 in VCaP cells.

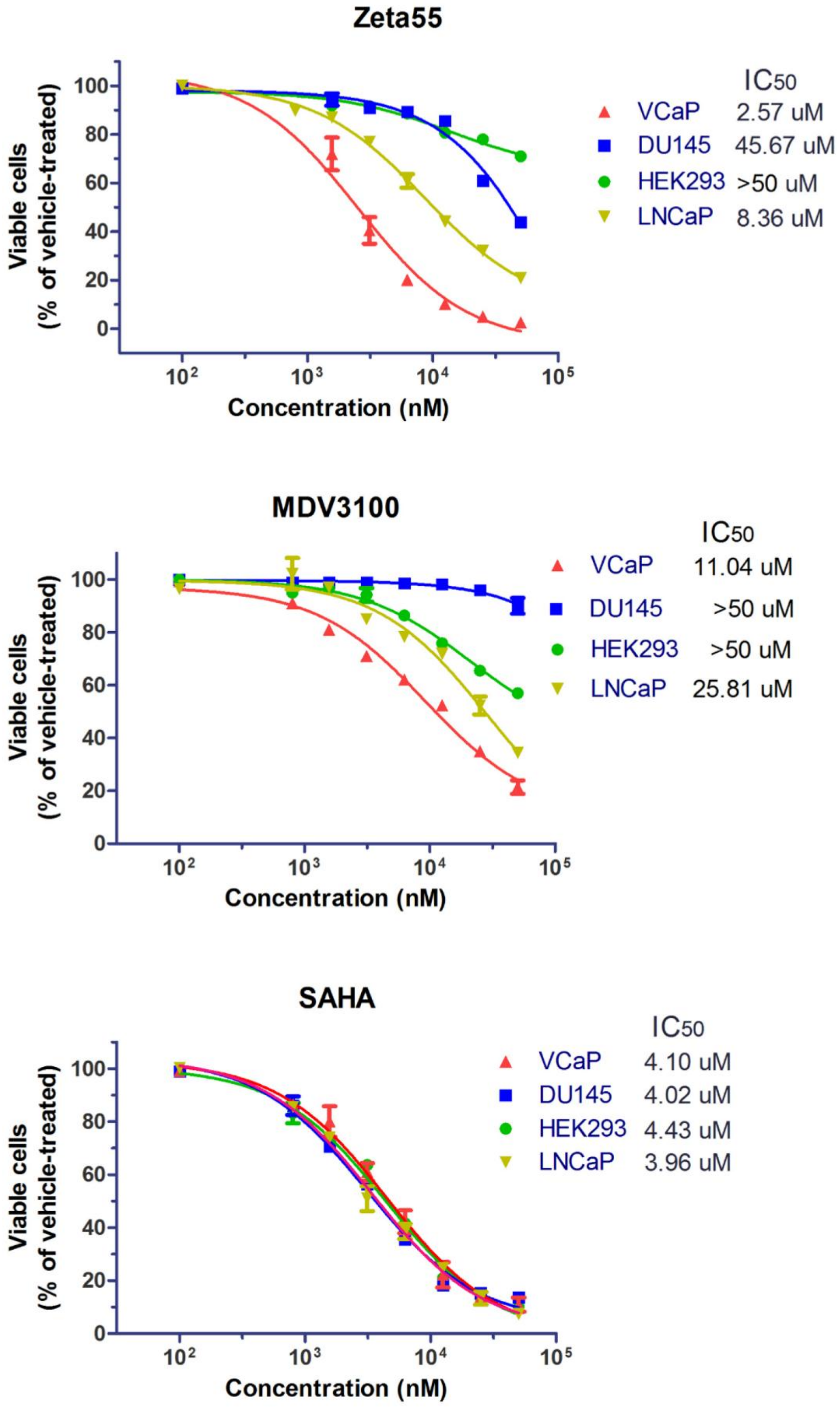

Supplementary Figure 5. IC_{50} values of Zeta55, MDV3100 and SAHA on the proliferation of VCaP, LNCaP, DU145 and HEK293 cells.

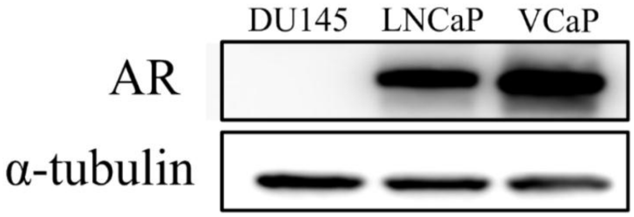

Supplementary Figure 6. AR expression in DU145, LNCaP and VCaP cells.
